# Mitochondrial transplantation ameliorates hippocampal damage following status epilepticus

**DOI:** 10.1002/ame2.12310

**Published:** 2023-02-03

**Authors:** Xiaoxia Jia, Qinghua Wang, Jianlun Ji, Wenchun Lu, Zhidong Liu, Hao Tian, Lin Guo, Yun Wang

**Affiliations:** ^1^ Jiangsu Key Laboratory of New Drug Research and Clinical Pharmacy Xuzhou Medical University Xuzhou China; ^2^ Psychology Laboratory, School of Management Xuzhou Medical University Xuzhou China; ^3^ Department of Pharmacy The Affiliated Hospital of Xuzhou Medical University Xuzhou China; ^4^ Agro‐Products Processing Research Institute Yunnan Academy of Agricultural Sciences Kunming China

**Keywords:** cognitive deficit, emotional disorders, hippocampal damage, mitochondrial transplantation, status epilepticus

## Abstract

**Background:**

Hippocampal damage caused by status epilepticus (SE) can bring about cognitive decline and emotional disorders, which are common clinical comorbidities in patients with epilepsy. It is therefore imperative to develop a novel therapeutic strategy for protecting hippocampal damage after SE. Mitochondrial dysfunction is one of contributing factors in epilepsy. Given the therapeutic benefits of mitochondrial replenishment by exogenous mitochondria, we hypothesized that transplantation of mitochondria would be capable of ameliorating hippocampal damage following SE.

**Methods:**

Pilocarpine was used to induced SE in mice. SE‐generated cognitive decline and emotional disorders were determined using novel object recognition, the tail suspension test, and the open field test. SE‐induced hippocampal pathology was assessed by quantifying loss of neurons and activation of microglia and astrocytes. The metabolites underlying mitochondrial transplantation were determined using metabonomics.

**Results:**

The results showed that peripheral administration of isolated mitochondria could improve cognitive deficits and depressive and anxiety‐like behaviors. Exogenous mitochondria blunted the production of reactive oxygen species, proliferation of microglia and astrocytes, and loss of neurons in the hippocampus. The metabonomic profiles showed that mitochondrial transplantation altered multiple metabolic pathways such as sphingolipid signaling pathway and carbon metabolism. Among potential affected metabolites, mitochondrial transplantation decreased levels of sphingolipid (d18:1/18:0) and methylmalonic acid, and elevated levels of D‐fructose‐1,6‐bisphosphate.

**Conclusion:**

To the best of our knowledge, these findings provide the first direct experimental evidence that artificial mitochondrial transplantation is capable of ameliorating hippocampal damage following SE. These new findings support mitochondrial transplantation as a promising therapeutic strategy for epilepsy‐associated psychiatric and cognitive disorders.

## Graphical abstract

Mitochondrial dysfunction is one of contributing factors in hippocampal damage caused by status epilepticus (SE). Mitochondrial replenishment by exogenous mitochondria improves the cognitive deficits, depressive and anxiety‐like behaviors following SE.

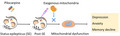

## 1 INTRODUCTION

Epilepsy is a chronic disease of the brain characterized by recurrent and spontaneous seizures that affects around 45 million people worldwide.^1^ Epileptic seizure, particularly status epilepticus (SE), can lead to hippocampal damage including gliosis, oxidative stress and neuronal loss in the brain.^2^ These pathophysiological events caused by SE not only increase the risk of subsequent seizures (i.e. epileptogenesis) but also contribute to the development of cognitive decline and emotional disorders.^3^, ^4^ Notably, although currently prescribed anticonvulsants are successful at terminating SE, numerous anticonvulsants are not capable of ameliorating pathophysiological events after SE.^5^ Therefore, there is an urgent need to develop novel therapies protecting against brain damage after SE.

Mitochondrial dysfunction is considered to be one of the contributing factors to many neurological diseases. Recently, growing evidence suggests that mitochondrial dysfunction not only occurs as a consequence of epileptic seizures, but is also a potential cause of secondary brain damage following epileptic seizures. For example, the expression of mitochondrial trafficking kinesin protein 1 (TRAK1) is decreased in temporal lobe epilepsy, and silencing of TRAK1 increases susceptibility to seizures.^6^ In contrast, inhibition of mitochondrial dihydroorotate dehydrogenase (DHODH) can decrease susceptibility to seizure by suppressing hippocampal CA3‐CA1 synaptic transmission and CA1 mean firing rate.^7^ Restoration of deficiencies in mitochondrial respiratory complex I exerts a protective effect in KA‐induced seizures.^8^ Therefore, mitochondrial components should receive urgent attention as potential therapeutic targets for epilepsy.

Notably, mitochondria are cellular organelles and it is thus also possible to transfer whole mitochondria intercellularly. In the brain, astrocytes can transfer their healthy mitochondria to adjacent neurons to protect against ischemic injury.^9^ In the peripheral system, adipocytes are able to transfer mitochondria to neighboring macrophages, and this process can be attenuated by obesity.^10^ Beyond endogenous mitochondrial transfer, the development of exogenous mitochondria transplantation (namely artificial mitochondrial transfer) has recently aroused considerable attention and interest. For instance, treatment using isolated mitochondria from N2a cells reduces reactive oxygen species (ROS) and apoptosis levels caused by hypoxia/reoxygenation.^11^ Treatment with exogenous healthy mitochondria can suppress the proliferation of hepatocellular carcinoma cells.^12^ Moreover, nasal administration of human mesenchymal stem cell‐derived mitochondria restores chemotherapy‐induced cognitive deficits in mice.^13^ Even more interestingly, mitochondria from soleus can also benefit functional recovery in rats suffering from traumatic spinal cord injury,^14^ suggesting a promising role for artificial mitochondrial transfer.

Based on the findings that mitochondrial dysfunction underlies SE‐induced brain damage, and that transplanting healthy mitochondria can rescue mitochondrial dysfunction and damaged cellular function, we investigated whether treatment with exogenous mitochondria would ameliorate SE‐induced hippocampal damage including neuronal loss, gliosis, cognitive deficit and emotional disorders. Our results provide new insights into the development of therapeutic strategies targeting epilepsy.

## 2 MATERIALS AND METHODS

### 2.1 Animal sourcing and ethics statement

Male C57BL/6J mice (22–25 g) were obtained from the Central Animal House, Xuzhou Medical University. Animals were housed in plastic cages (5 per cage) in a specific pathogen‐free room with a light/night (12/12 h) cycle and a temperature range of 20–26°C. During the experiments, the animals had free access to food and water. All animal care and experimental protocols were approved by the Institutional Animal Care and Use Committee of Xuzhou Medical University and were in accordance with the ARRIVE guidelines (Animals in Research: Reporting In Vivo Experiments), and the *Guidelines for the Care and Use of Laboratory Animals* (Chinese‐National‐Research‐Council, 2006).^15^

### 2.2 Isolation of mitochondria

Mitochondria were isolated using the Tissue Mitochondria Isolation Kit (Beyotime, Shanghai, China) according to the manufacturer's protocol. Briefly, normal mice were decapitated after anesthesia (pentobarbital sodium, 150 mg/kg, i.p.). The hippocampus was dissected out and homogenized using mitochondrial lyses buffer A. The homogenate was centrifuged at 1000*g* for 5 min at 4°C, and the supernatant was centrifuged again at 3500*g* for 10 min at 4°C. The pellet was resuspended in stock buffer. The JC‐1 assay kit (Beyotime, Shanghai, China) was used to determine mitochondrial membrane potential. The fluorescence intensity was detected using a multifunctional microplate reader (excitation wavelength: 485 nm, emission wavelength: 590 nm). Mitotracker red CMXRos (0.2 μmol/L; Invitrogen, Cambridge, MA; 37°C, 30 min) was used to label mitochondria.

### 2.3 Pilocarpine‐induced SE

Animals were first administered with scopolamine methyl nitrate (2 mg/kg; Sigma, St Louis, Missouri) to antagonize the peripheral effects of pilocarpine. After 30 min, pilocarpine hydrochloride (320 mg/kg; Sigma) was injected to induce SE. The severity of seizures was rated according to the scales proposed previously.^16^, ^17^ Two hours after the first episode of level 4 or 5 seizures, diazepam (10 mg/kg, i.m.) was injected to stop seizure activity. For mitochondrial treatment, the protein concentration of fresh mitochondria was assessed using a BCA assay kit (Beyotime, Shanghai, China), and the mitochondria were then diluted with normal saline. Exogenous fresh mitochondria were administered once by the tail vein injection (1 mg/kg) 1 hour after diazepam using a published protocol.^18^ The same volume of inactivated mitochondria was using as a negative control.

### 2.4 Behavioral tests

The behavioral tests were implemented 7–10 days after SE. The animals' memory function was determined using the novel object recognition test.^19^ The recognition index used was defined as: time consumed in the new object/(time consumed in the new object + time consumed in the familiar object).^20^ Depressive‐ and anxiety‐like behaviors were evaluated using the tail suspension test (TST) and the open field test (OFT), respectively. The time spent immobile in TST, and time in the central zone in OFT were calculated using the ANY‐maze tracking system (Stoelting Co, IL).^21^

### 2.5 Immunofluorescence

Animals were anesthetized (pentobarbital sodium, 150 mg/kg, i.p.) and transcardially perfused with ice‐cold normal saline, and subsequently with 4% paraformaldehyde (PFA). The brains were extracted and immersed in 4% PFA for 12 h post‐fixation, and then in a 30% sucrose solution overnight. Mounted sections 30 μm thick were permeabilized with 0.01 M PBS containing 0.1% Triton X‐100, and blocked with 10% goat serum. The sections were incubated with primary rabbit anti‐GFAP antibodies (1:300, Cell Signaling Technology, Boston, MA, USA), anti‐Iba1 (1:300, Wako, Osaka, Japan), anti‐MAP2 (1:300, Proteintech Group, USA). After incubation with the respective secondary antibody, DAPI (Sigma‐Aldrich, St Louis, MO, USA) was used to label nuclei. Images of immunostaining were acquired with a fluorescence microscope (Leica, Solms, Germany) and the number of positive cells was manually counted.

### 2.6 ROS determination

Hippocampuses were cut into smaller pieces (approximately 1 mm^2^) and digested with 0.25% trypsin for 20 min at 37°C. The undigested pieces were twisted gently in 200 mesh net. After filtration, the suspension was centrifuged at 1500*g* for 5 min and the pellets were collected. The deposited cells were washed with PBS and incubated with 10 μM 5‐(and‐6)‐carboxy‐2′,7′‐dichlorodihydrofluorescein diacetate (DCFH‐DA) at 37°C for 20 min (Nanjing Jiancheng Bioengineering Institute, Nanjing, China).

### 2.7 Metabolomics

Hippocampuses were homogenized with 200 μL H_2_O and five ceramic beads, and 800 μL methanol/acetonitrile (1:1, v/v) was then added. After centrifugation (20 min, 14 000*g*, 4°C), the supernatant was dried in a vacuum centrifuge. The samples were then re‐dissolved in 100 μL acetonitrile/water (1:1, v/v) solvent and centrifuged at 14 000*g* at 4°C for 15 min. The resulting supernatant was injected. Quality control (QC) samples were prepared by pooling 10 μl of each sample. Analysis was performed using an UHPLC system (Vanquish UHPLC, Thermo) coupled to a quadrupole time‐of‐flight system (AB Sciex TripleTOF 6600) by Shanghai Applied Protein Technology Co., Ltd. Compound identification of metabolites was performed by comparing the accuracy m/z value (<10 ppm) and MS/MS spectra with an in‐house database (Shanghai Applied Protein Technology). The variable importance in the projection (VIP) value of each variable in the OPLS‐DA model was calculated to indicate its contribution to the classification. VIP >1 and *P* value <0.05 were classified as ‘changing’ metabolites.

### 2.8 Statistical analysis

Data were expressed as mean ± SEM. One‐way ANOVA analysis followed by Tukey's post hoc test was used for multiple comparison. Statistical significance levels were set at *p* < 0.05 (*) and *p* < 0.01 (**). Data were analyzed using the GraphPad Prism software (Version 8.0, GraphPad Software, Inc. La Jolla, CA, USA).

## 3 RESULTS

### 3.1 Properties of isolated mitochondria

**FIGURE 1:**
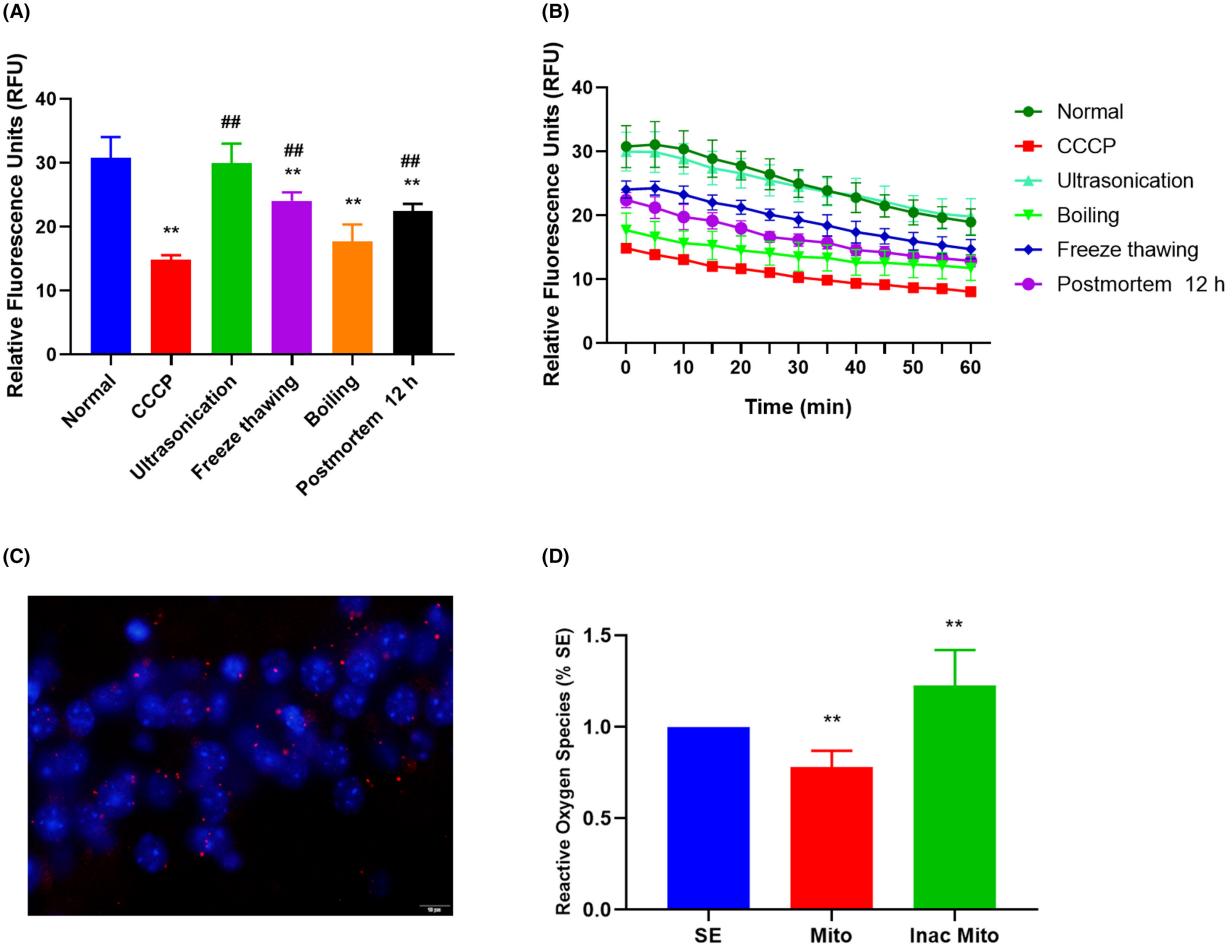
Properties of isolated mitochondria. (A) Membrane potential of isolated mitochondria assessed by JC‐1 staining. Isolated mitochondria were divided into six groups (*N* = 4 in each group) according to treatment: normal and carbonyl cyanide 3‐chlorophenylhydrazone (CCCP) positive controls; freeze thawing for 30 min; boiling for 10 min; ultrasonication for 30 min; and extraction from postmortem mice 12 h after death. *F* (5, 18) = 32.05, *p* < 0.0001. **p < 0.01, compared with the normal group. ^##^ *p* < 0.01, compared with the CCCP group. (B) The dynamic membrane potential of isolated mitochondria within 1 hour at room temperature. (C) Immunofluorescence staining in the hippocampus following peripheral injection of exogenous mitochondria in the SE group. Mitochondria were labeled by MitoTracker Red, and the nucleus was stained using DAPI. Scale = 10 μm. (D) The effect of exogenous mitochondria on SE‐induced ROS production. *N* = 10 in each group, *F* (2, 27) = 30.53, *p* < 0.0001. **p < 0.01, compared with the SE group. Data were expressed as means ± SEM and analyzed using one‐way ANOVA.

We first evaluated the integrity of isolated mitochondria using detection of membrane potential (ψm) by JC‐1 staining. The relative fluorescence of mitochondria in the normal group was 30.78 ± 3.28 relative fluorescence units (RFU), and in the carbonyl cyanide 3‐chlorophenylhydrazone (CCCP) positive control group it was 14.84 ± 0.72 RFU. We further determined the stability of isolated mitochondria. The results showed that freeze thawing and boiling but not ultrasonication can significantly reduce the mitochondrial membrane potential of isolated mitochondria compared to the normal control group. However, isolated mitochondria undergoing freeze thawing and mitochondria from postmortem mice (12 h after death) still retained higher activity than the CCCP group (Figure 1A). Moreover, the membrane potential of isolated mitochondria decreased over time, suggesting the need for freshly purified mitochondria (Figure 1B).

Exogenous mitochondria are believed to be capable of penetrating the blood–brain barrier.^22^ In the present study, taking advantage of MitoTracker Red CMXRos (Mito Red) staining, we confirmed that the exogeneous mitochondria could cross the blood–brain barrier (Figure 1C). To discover whether replenishment artificial mitochondrial treatment could improve SE‐induced mitochondrial dysfunction, we assessed changes in ROS. As shown in Figure 1D, exogeneous mitochondria treatment significantly reduced SE‐produced ROS. However, it is noteworthy that mitochondria inactivated by boiling failed to alleviate and even accelerated the level of ROS. These results suggested that our isolated mitochondria were functional, and treatment with exogenous mitochondria was able to ameliorate SE‐induced mitochondrial dysfunction.

### 3.2 The effect of exogenous mitochondria on cognitive decline and depression‐ and anxiety‐like behavior in post‐SE mice

**FIGURE 2:**
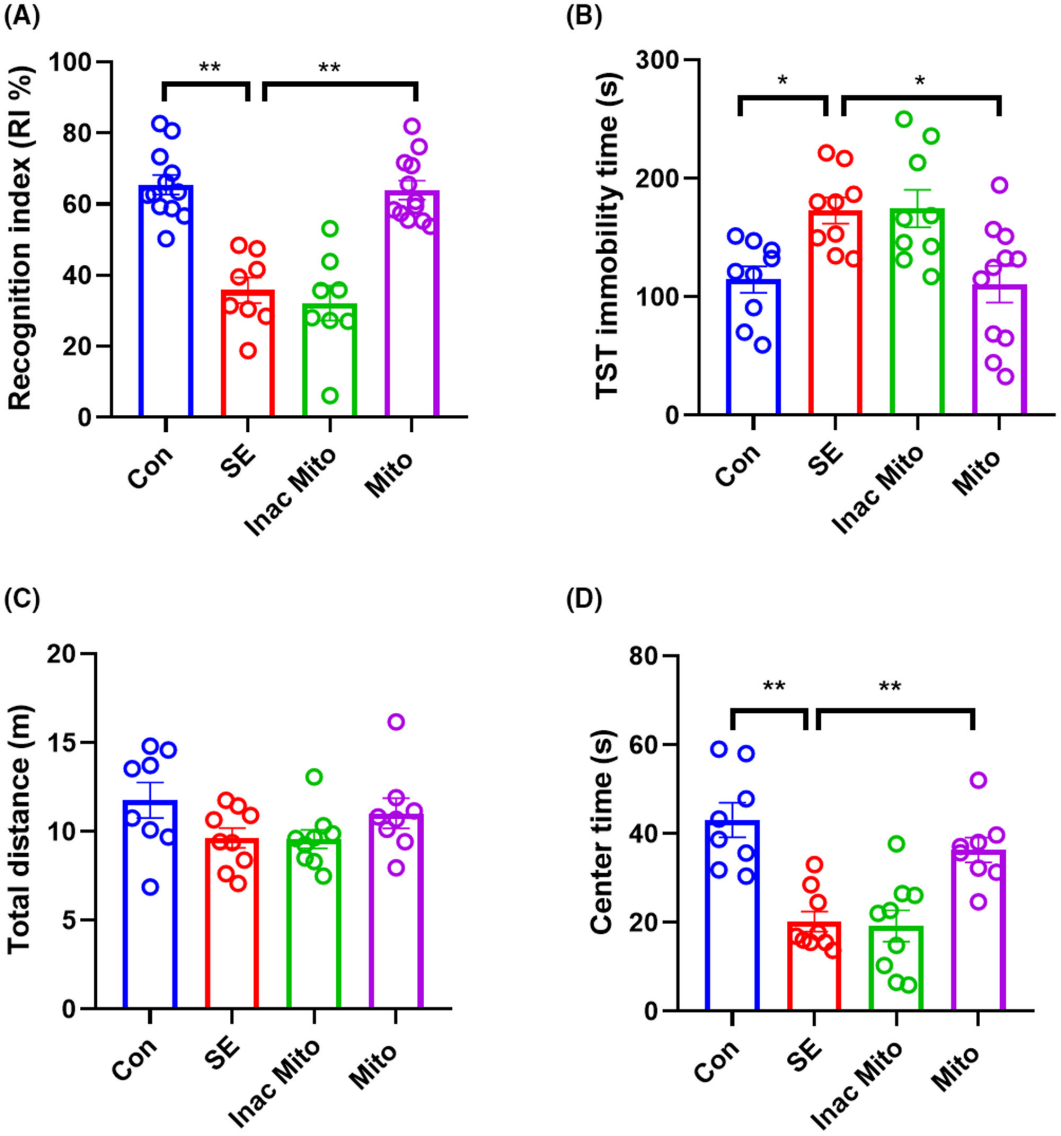
The effect of exogenous mitochondria on the cognitive decline and emotional disorders. (A) Novel object recognition test (NORT). (B) Tail suspension test (TST). (C) Total distances in the open field test (OFT). (D) Time in the central area in the OFT. A single injection of exogenous mitochondria (1 mg/kg) was administered after SE termination. Behaviors were determined on days 7–10 after SE. Data were expressed as means ± SEM and analyzed using one‐way ANOVA followed by Tukey's multiple‐comparison test. *N* = 8–10 in each group. In NORT, *F* (3, 36) = 27.20, *p* < 0.0001. In TST, *F* (3, 34) = 6.522, *p* < 0.01. In the center of OFT, *F* (3, 30) = 13.94, *p* < 0.0001. *p < 0.05, **p < 0.01.

Cognitive deficit and mood dysfunction (such as depression and anxiety) are considered to be the most common complications of epilepsy. Next we sought to determine the effect of artificial transplantation of mitochondria on these behaviors after SE. In the NORT (Figure 2A), SE caused a decrease in recognition index, whereas the exogenous injection of normal mitochondria but not inactivated mitochondria improved the recognition index. Meanwhile, in the TST (Figure 1B), the immobility time of the SE group was significantly elevated compared to the control group, and normal but not inactivated mitochondria decreased the immobility time. Similarly, in the open field test (Figure 2C,D), exogenous mitochondria significantly rescued the lower time spent in the central zone caused by SE, but did not alter total distances. Collectively, these results demonstrated that artificial mitochondrial transplantation could ameliorate the cognitive impairment, epileptic depression and anxiety state caused by SE.

### 3.3 The effect of exogenous mitochondria on neuronal loss in hippocampus after SE

**FIGURE 3:**
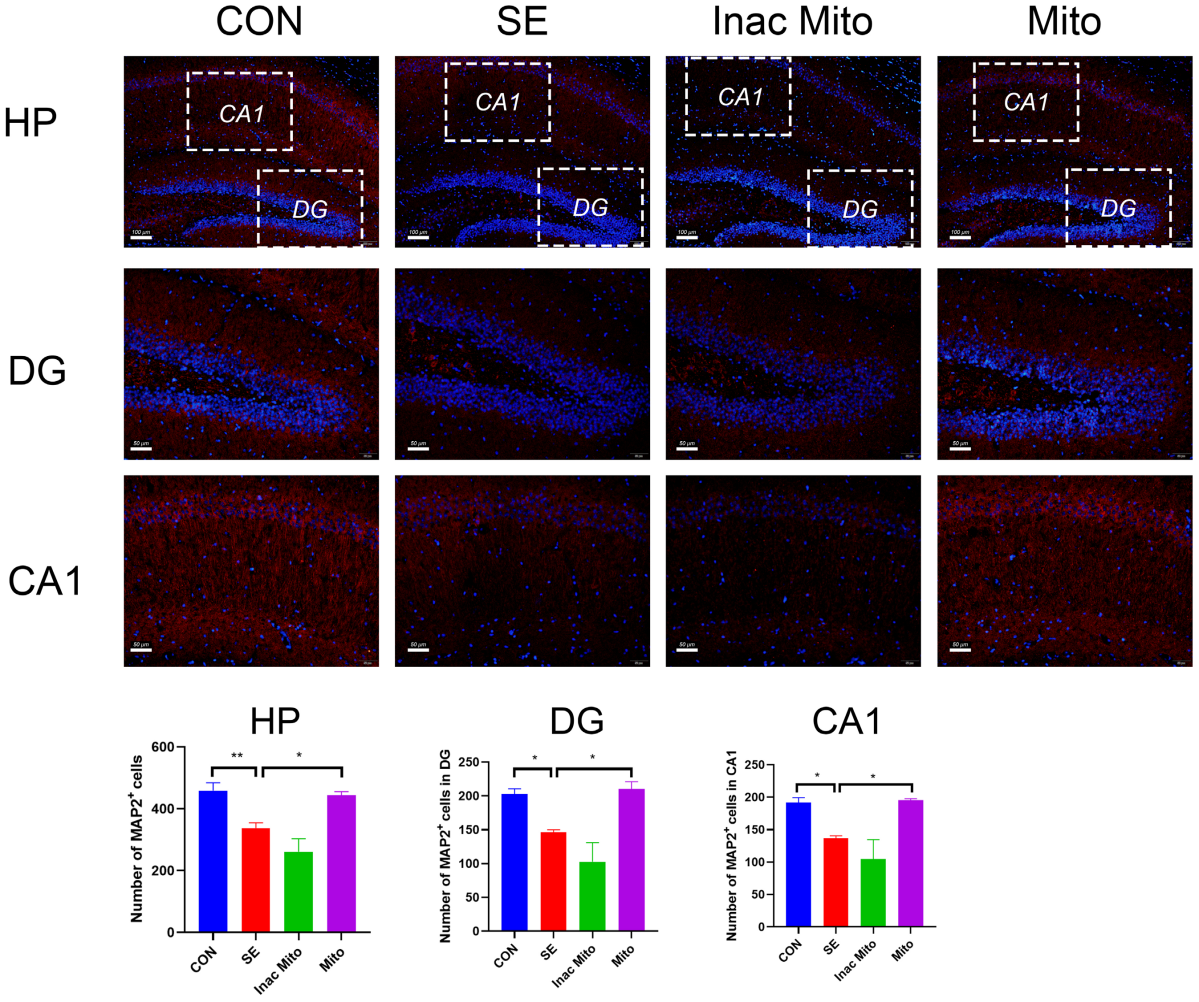
The effect of exogenous mitochondria on neuronal loss in hippocampus after SE. Neurons were labeled with anti‐MAP2 and nuclei were stained using DAPI. Immunofluorescence staining is shown in the HP (hippocampus, scale = 100 μm; top), DG (scale = 50 μm; middle), and CA1 (scale = 50 μm; bottom). Data were expressed as means ± SEM and analyzed using one‐way ANOVA followed by Tukey's multiple‐comparison test. *N* = 5–6 in each group. For neuronal loss in HP, *F* (3, 19) = 13.36, *p* < 0.0001. For neuronal loss in DG, *F* (3, 19) = 12.01, *p* < 0.0001. For neuronal loss in CA1, *F* (3, 19) = 9.873, *p* < 0.001. *p < 0.05, **p < 0.01.

SE induced‐memory deficits and mood disorders are generally accompanied by various kinds of cellular abnormality, including neuronal loss and glial activation. In the present study, MAP2 immunostaining showed that the number of hippocampal neurons was significantly reduced in the SE group compared to the control group (Figure 3).^23^ Moreover, neuronal loss occurred in sub‐regions of the hippocampus such as dentategyrus (DG) and the CA1 pyramidal cell layer. After treatment with exogenous mitochondria, neuronal loss was attenuated. In contrast, inactivated mitochondria are unable to protect against neuronal loss. These data revealed that artificial mitochondria transplantation was able to mitigate neuronal loss caused by SE.

### 3.4 The effect of exogenous mitochondria on the proliferation of microglia and astrocytes after SE

**FIGURE 4:**
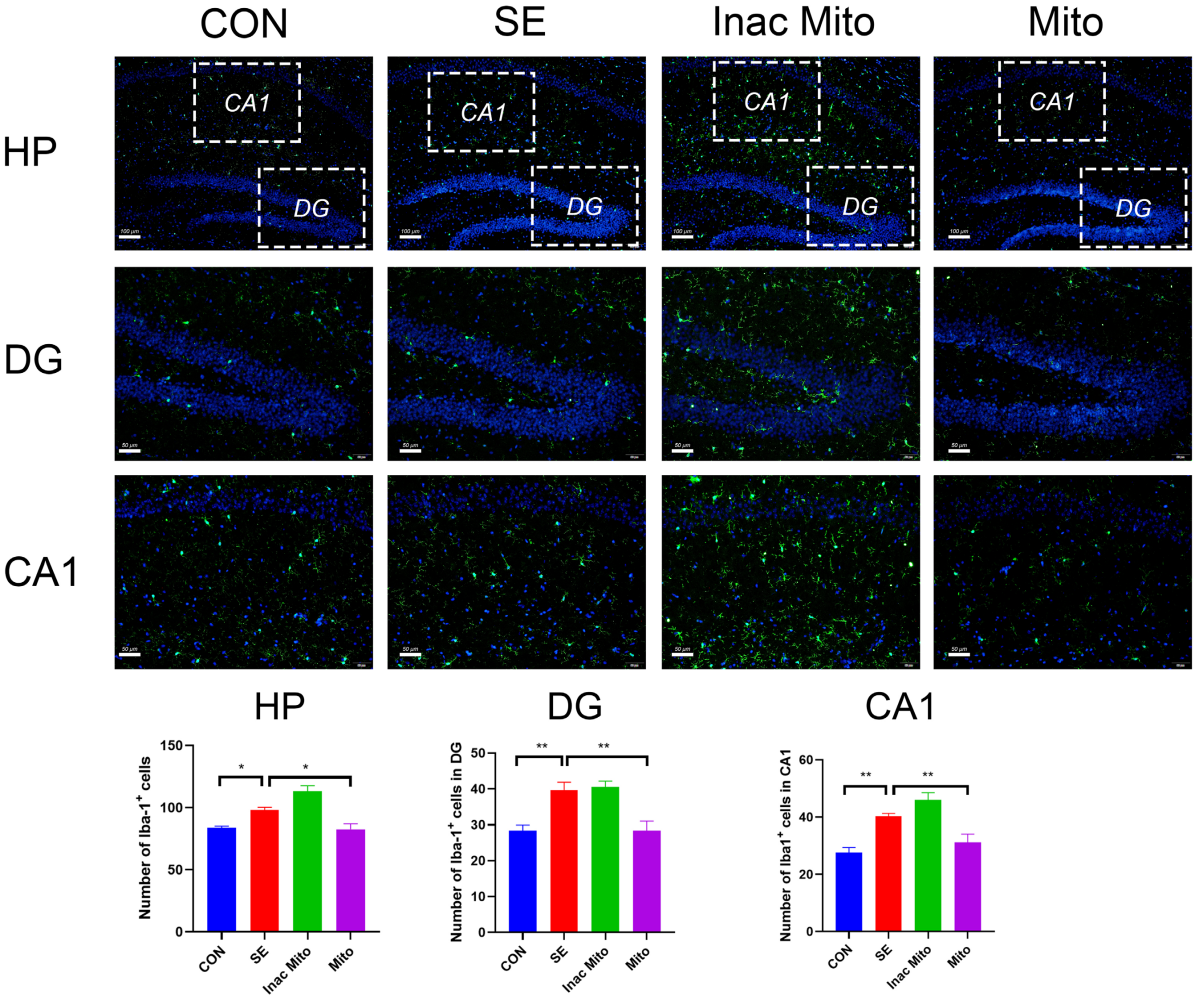
The effect of exogenous mitochondria on the activated microglia in hippocampus after SE. Microglia were labeled with anti‐Iba1 and nuclei were stained using DAPI. Immunofluorescence staining is shown in the HP (hippocampus, scale = 100 μm; top), DG (scale = 50 μm; middle), and CA1 (scale = 50 μm; bottom). Data are expressed as means ± SEM and analyzed using one‐way ANOVA followed by Tukey's multiple‐comparison test. *N* = 6 in each group. For proliferation of microglia in HP, *F* (3, 20) = 17.79, *p* < 0.0001. For proliferation of microglia in DG, *F* (3, 20) = 15.32, *p* < 0.0001. For proliferation of microglia in CA1, *F* (3, 20) = 10.30, *p* < 0.001. *p < 0.05, **p < 0.01.

**FIGURE 5:**
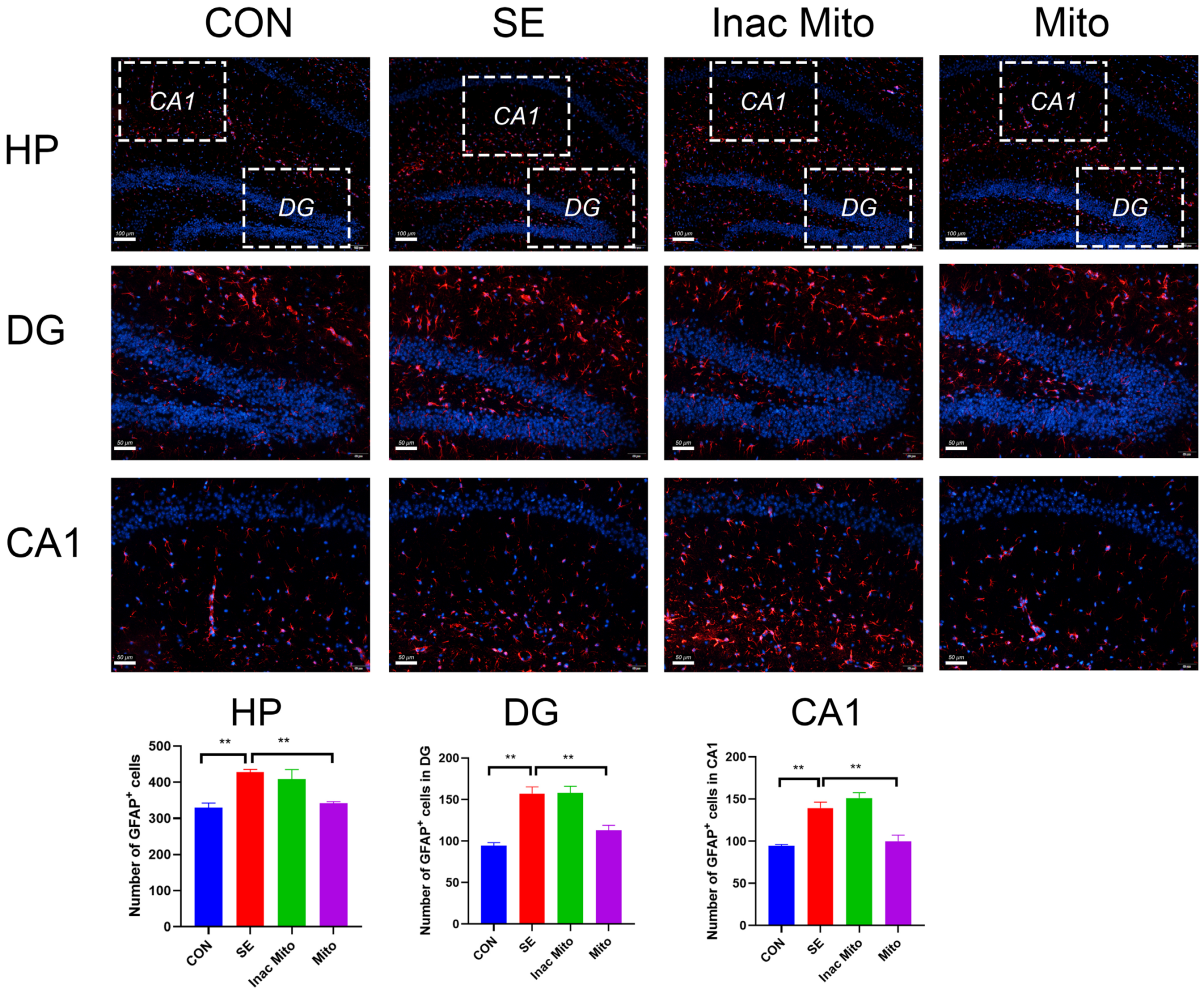
The effect of exogenous mitochondria on the activated astrocyte in hippocampus after SE. Astrocytes were labeled with anti‐GFAP and nuclei were stained using DAPI. Immunofluorescence staining is shown in the HP (hippocampus, scale = 100 μm; top), DG (scale = 50 μm; middle), and CA1 (scale = 50 μm; bottom). Data are expressed as means ± SEM and analyzed using one‐way ANOVA followed by Tukey's multiple‐comparison test. *N* = 6 in each group. For proliferation of astrocytes in HP, *F* (3, 20) = 9.521, *p* < 0.001. For proliferation of astrocytes in DG, *F* (3, 20) = 21.55, *p* < 0.0001. For proliferation of astrocyte in CA1, *F* (3, 20) = 22.31, *p* < 0.0001. *p < 0.05, **p < 0.01.

Glial cells including microglia and astrocytes are crucial regulators of the immune response in the brain. The activation of microglia and astrocytes contributes to neuronal loss and plasticity in epilepsy.^24^ Therefore, we examined the proliferation of microglia and astrocytes, which is one of the characteristics of glial activation. As shown in Figure 4, the number of Iba1^+^ microglia in either DG or CA1 was significantly increased in the SE group compared to the control groups. Similarly, the number of cells staining positive with GFAP was also augmented after SE (Figure 5). Correspondingly, exogenous mitochondria treatment significantly reduced the proliferation of microglia and astrocytes, suggesting that artificial mitochondria transplantation can inhibit glial activation after SE.

### 3.5 The effect of exogenous mitochondria on metabonomic profiles in hippocampus after SE

**FIGURE 6:**
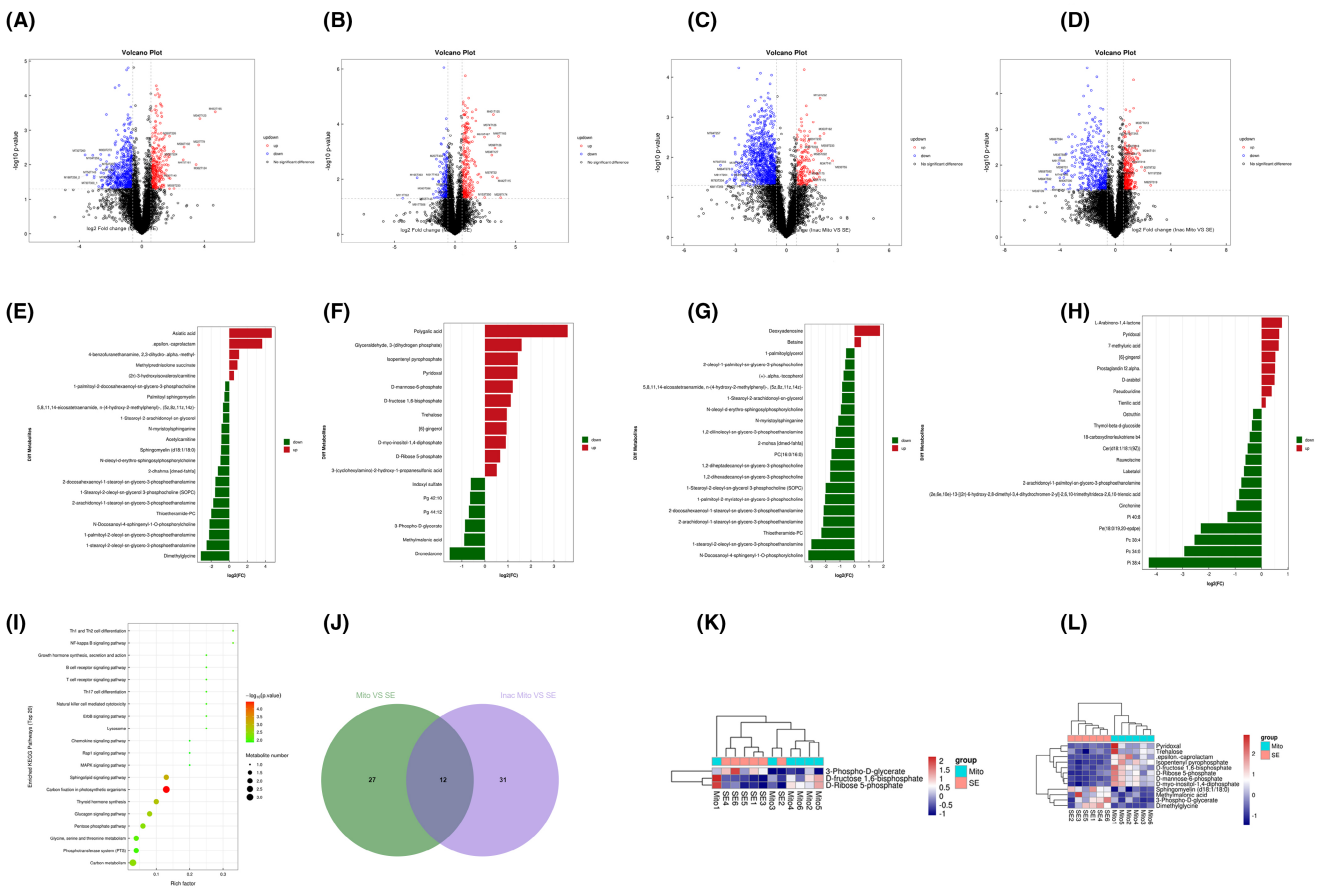
The effect of exogenous mitochondria on metabonomic profiles in hippocampus after SE. (A and B) Model of identified metabolites with positive ions (A) and negative ions (B) that differed between the mitochondrial transplantation and SE groups. (C and D) Model of identified metabolites with positive ions (C) and negative ions (D) that differed between the exogenous inactivated mitochondria and SE groups. (E and F) Metabolites with positive ions (E) and negative ions (F) showing significant differences between the mitochondrial transplantation and SE groups. (G) and (H), Metabolites with positive ions (G) and negative ions (H) showing significant differences between the exogenous inactivated mitochondria and SE groups. (I) KEGG pathway analysis of the metabolites that differed between the mitochondrial transplantation and SE groups. (J) The number of changed metabolites shared by both the inactivated and functional mitochondria groups. (K and L) Heat maps of potential metabolites.

In the present study, 39 metabolites were differentially expressed between the group treated with exogenous mitochondria and the SE group (Figure 6 and Tables S1 and S2). Among these metabolites, 16 metabolites were upregulated and 23 metabolites were downregulated. In addition, biological pathway analysis indicated that the metabolites identified as underlying the therapeutic action of mitochondria are enriched in multiple metabolism pathways including carbon fixation in photosynthetic organisms, the sphingolipid signaling pathway, and carbon metabolism (Figure 6I). To further exclude metabolites inconsistent with the therapeutic effect, we also identified the metabolites that differed between the group treated with inactivated mitochondria and the SE group. As shown in Figure 6J, there are 12 metabolites showing the same profiles in both inactivated and functional mitochondria groups. Among the rest of the metabolites associated with the exogenous mitochondria treatment group, some potential metabolites such as glyceraldehyde, fructose‐1,6‐bisphosphate 3‐(dihydrogen phosphate), isopentenyl pyrophosphate, D‐mannose‐6‐phosphate, D‐fructose 1,6‐bisphosphate, and (2r)‐3‐hydroxyisovaleroylcarnitine were upregulated, while other potential metabolites such as sphingolipid (d18:1/18:0), methylmalonic acid, 3‐phospho‐D‐glycerate, and dimethylglycine were downregulated (Figure 6K,L).

## 4 DISCUSSION

Mitochondria are attracting growing attention as a promising therapeutic strategy in epilepsy. Here, we investigated the effect of the transplantation of mitochondria on SE‐associated hippocampal neuropathology. The results of the present study showed that peripheral administration of isolated mitochondria could cross the blood–brain barrier and reduce SE‐induced ROS. At the cellular level, exogenous mitochondria blunted the proliferation of microglia and astrocytes, as well as loss of neurons in hippocampus. In addition, exogenous mitochondria improved the cognitive deficits depressive‐ and anxiety‐like behaviors induced by SE. To the best of our knowledge these findings provide the first direct experimental evidence that artificial mitochondria transplantation is capable of ameliorating hippocampal damage following SE.

The hippocampus is a crucial brain area for learning, memory and mood. On the one hand, hippocampal injury following SE causes epileptogenesis and leads to subsequent seizures. On the other hand, hippocampal injury can manifest as memory deficits and mood dysfunction. In the present study, glial proliferation and neuronal loss – critical pathological events in hippocampal damage – were observed in hippocampus after SE. Reactive microglia in the hippocampus have been found as early as 1 day, and even 4 h, after pilocarpine‐induced seizures, and the reactive state can remain for at least 3–5 days.^25^, ^26^ Meanwhile, reactivated astrocytes are increased at 24 h, whereas the neuronal damage is found as early as 3 h after SE.^27^ Activation of microglia after SE can release pro‐inflammatory cytokines, leading to neuronal damage, and also enhance the activation of adjacent astrocytes, further amplifying neuronal injury.^28^ There is a growing body of research indicating that activation of microglia‐astrocyte crosstalk contributes to neuronal damage in epilepsy.^29^ Indeed, this kind of neuroinflammatory process caused by microglia‐astrocyte crosstalk is thought to be a contributory factor underlying consequent cognitive decline and mood dysfunctions.^30^, ^31^

Further, as intracellular ‘power plants’, mitochondria are well documented to transfer between cells in the brain. For example, microglia can release dysfunctional mitochondria to trigger neuronal damage, while astrocytes can transfer functional mitochondria to support neuronal survival.^9^, ^32^ Although the mechanism(s) by which mitochondria enter recipient cells are not clear, multiple pathways for mitochondrial transfer have been described, such as tunneling nanotubes (TNTs), extracellular vesicles (EVs), and endocytosis.^33^, ^34^, ^35^ In the present study, peripheral treatment with exogenous mitochondria resulted in wide distribution of mitochondria throughout the hippocampus, suggesting an effective route of administration. In addition, isolated mitochondria are relatively stable, even after ultrasonication and freeze thawing. More importantly, isolated mitochondria originating from varying sources including autologous, allogenic, and xenogeneic sources, have recently been used in mitochondrial transplantation,^36^ with promising implications. However, we still do not yet know the overall distribution and “pharmacokinetics” of exogenous mitochondria in the brain and in our study electroencephalogram (EEG) recordings were not used to score seizures induced by pilocarpine. Therefore, more intensive studies should be carried out to address these problems in the future.

Mitochondrial functions in epilepsy are complicated. In the present study, replenishment of dysfunctional mitochondria by exogenous transplantation not only diminishes SE‐induced ROS production but also alters the expression and metabolic pathways of numerous metabolites. For example, mitochondrial transplantation decreased the level of sphingomyelin (d18:1/18:0) compared with the SE group. In a pentylenetetrazol (PTZ) kindling epilepsy model, sphingomyelins are elevated in the brain.^37^ A study of cognitive impairment disease showed that the level of sphingolipid (d18:1/18:0) is increased in cerebrospinal fluid of Alzheimer's disease patients.^38^ Sphingomyelins are degraded with hydrolyzation dependent on different sphingomyelinases. A mammalian neutral sphingomyelinase located on mitochondria has been identified,^39^ and deficiency of sphingomyelins is reported to give rise to microglial proliferation,^40^ suggesting a key role of sphingomyelins in epilepsy. Mitochondrial transplantation can also reduce the level of methylmalonic acid. Methylmalonic acid can directly cause oxidative damage in synaptosomes,^41^ while accumulation of methylmalonic acid also results in methylmalonic acidemia with neurologic symptoms including seizures and cognitive impairments.^42^ Mitochondrial transplantation also affects carbon metabolism pathways including D‐ribose 5‐phosphate, D‐fructose 1,6‐bisphosphate, and 3‐phospho‐D‐glycerate. D‐fructose 1,6‐bisphosphate, for instance, a glycolytic intermediate inhibiting glycolysis, has anticonvulsant and anti‐inflammatory effects and reduces inflammatory pain‐like behavior,^43^, ^44^ suggesting an extensive role of mitochondrial transplantation.

In summary, we have demonstrated that exogenous mitochondria can ameliorate the memory decline and mood dysfunctions (depressive‐ and anxiety‐like behaviors) following SE. Exogenous mitochondria inhibit the loss of neurons and the activation of microglia and astrocytes, as well as affecting a multitude of metabolites in the hippocampus after SE injury. The present study is the first to support mitochondrial transplantation as a promising strategy for effectively treating epilepsy‐associated psychiatric and cognitive disorders.

## AUTHOR CONTRIBUTIONS

**Xiaoxia Jia:** Investigation. **Qinghua Wang:** Investigation. **Jianlun Ji:** Investigation. **Wenchun Lu:** Investigation. **Zhidong Liu:** Investigation. **Hao Tian:** Supervision, Writing – Review & Editing. **Lin Guo:** Investigation, Conceptualization, Funding acquisition, Writing – Original draft. **Yun Wang:** Conceptualization, Funding acquisition, Supervision, Writing – Review & Editing. All authors read and approved the final manuscript.

## CONFLICT OF INTEREST STATEMENT

All authors declare that they have no conflicts of interest. Yun Wang is an Editorial Board member of AMEM and a co‐author of this article. To minimize bias, he was excluded from all editorial decision‐making related to the acceptance of this article for publication.

## CONSENT TO PARTICIPATE

Not applicable.

## CONSENT FOR PUBLICATION

Not applicable.

## ETHICS APPROVAL

This study was performed in line with the ‘ARRIVE’ guidelines (Animals in Research: Reporting In Vivo Experiments), and the Guidelines for the Care and Use of Laboratory Animals (Chinese‐National‐Research‐Council, 2006). Approval was granted by the Ethics Committee of Xuzhou Medical University (No. 202205A423).

## Supporting information


Table S1.

Table S2.

## Data Availability

The data that support the findings of this study are available from the corresponding author, upon reasonable request.
